# Open source GIS for HIV/AIDS management

**DOI:** 10.1186/1476-072X-7-53

**Published:** 2008-10-22

**Authors:** Bas Vanmeulebrouk, Ulrike Rivett, Adam Ricketts, Melissa Loudon

**Affiliations:** 1 Centre for Geo-Information, Wageningen University and Research Centre, Wageningen, the Netherlands; 2 Department of Civil Engineering, University of Cape Town, Cape Town, South Africa; 3 Spatial Dimension, Cape Town, South Africa

## Abstract

**Background:**

Reliable access to basic services can improve a community's resilience to HIV/AIDS. Accordingly, work is being done to upgrade the physical infrastructure in affected areas, often employing a strategy of decentralised service provision. Spatial characteristics are one of the major determinants in implementing services, even in the smaller municipal areas, and good quality spatial information is needed to inform decision making processes. However, limited funds, technical infrastructure and human resource capacity result in little or no access to spatial information for crucial infrastructure development decisions at local level.

This research investigated whether it would be possible to develop a GIS for basic infrastructure planning and management at local level. Given the resource constraints of the local government context, particularly in small municipalities, it was decided that open source software should be used for the prototype system.

**Results:**

The design and development of a prototype system illustrated that it is possible to develop an open source GIS system that can be used within the context of local information management. Usability tests show a high degree of usability for the system, which is important considering the heavy workload and high staff turnover that characterises local government in South Africa. Local infrastructure management stakeholders interviewed in a case study of a South African municipality see the potential for the use of GIS as a communication tool and are generally positive about the use of GIS for these purposes. They note security issues that may arise through the sharing of information, lack of skills and resource constraints as the major barriers to adoption.

**Conclusion:**

The case study shows that spatial information is an identified need at local level. Open source GIS software can be used to develop a system to provide local-level stakeholders with spatial information. However, the suitability of the technology is only a part of the system – there are wider information and management issues which need to be addressed before the implementation of a local-level GIS for infrastructure management can be successful.

## Background

### HIV/AIDS in South Africa

South Africa has one the largest HIV-positive populations in the world – an estimated 5.41 million people were living with HIV in 2006 [1], which translates to a prevalence rate of about 10%. Antiretroviral treatment (ART) can improve the quality of life and life expectancy of HIV-positive people, delaying the progression from HIV to AIDS. In response to the epidemic, the Department of Health began a national rollout of antiretroviral treatment in 2004 [2]. While progress has been made, the scale of the rollout has proved challenging in the resource-limited public health sector, and in 2006 only 32% of those in need of treatment were receiving it [2]. Efforts to improve the reach of treatment programmes are ongoing, although the public health sector is overburdened and there are many challenges to be overcome before these are fully effective.

It is widely accepted that increased access to basic services (for example, clean water, sanitation, public transport and education) can improve a community's resilience to HIV/AIDS [3-7].

Education and better quality of life can result in fewer infections, and those that are infected will be healthier for longer. This, in addition to the obvious advantages for patients, carers and families as well as worker productivity, also reduces the immediate burden on treatment sites. Work is therefore being done to upgrade infrastructure in affected areas, and planning for the strategic expansion of water works, schools and transport networks has become one of the highest priorities of local government. To accomplish this task, GIS is used by provinces and large municipalities to strategically assess needs across South Africa (see for instance [8]). Spatial characteristics, such as topography or the geographical dispersion of population are a major determinant of whether an area does or does not receive services, even in the smaller municipal areas. However, at local level of previously disadvantaged communities, there is little or no access to such information [9].

### GIS and health

The use of GIS within health may be categorised into two distinctive areas of health [10,11]:

• epidemiology

• health care.

Epidemiology is the study of the occurrence of disease, especially in relation to environmental features. This is an area in which the traditional analytical tools of GIS are employed a great deal [12]. The use of GIS in health care is concerned with factors such as hospital and clinic placement, accessibility to these and other services contributing to the health of people. The system described here aims to contribute to the second category.

Studies related to health care planning within the developing world generally describe issues around accessibility to health services. Investigations into accessibility are provided through distance measures in relation to health care reform and the provision of services to those in need in Costa Rica [13], the effects of physical accessibility in mountainous areas in Andean Bolivia [14], and the identification of areas with minimal access to health services in India [15]. In South Africa, GIS has been used to estimate travel times to clinics, quantifying physical accessibility and providing comparisons for varying urban landscapes [16].

Direct research on health services and the management thereof appears lacking within the developing world. An exception is presented by Deshpande et al. [17] illustrating the importance of effective data provision through using GIS to determine the number of private health practitioners in India. Research based in the developed world more effectively illustrates the potential for GIS to be used in the management of health systems and infrastructure, but also highlights a number of shortcomings and challenges in the incorporation of GIS in health settings [18-20].

Many reasons are offered for the lack of incorporation of GIS technologies. The majority of these are concerned with the end-users of GIS and a lack of consideration of their needs [19,20]. Another reason for GIS not being implemented and used to the same extent as other application software is the time required to learn and understand its functionality [18,20].

Furthermore, GIS may be viewed as an undemocratic technology [21], as it further broadened gaps between the powerful and less empowered people through differential access to data and technology. Through GIS, those in power can use the surveillance capabilities to which other sectors of the population do not have access [22]. Participatory GIS is a field of study and application that has emerged from the criticism of GIS and power structures. Participatory GIS or community integrated GIS refers to a methodology that allows systems (GIS) to address the needs of people who are concerned with participation in decision making [23].

### GIS and local communities

Community involvement in the planning process is not only a fundamental requirement if development is to be sustainable in the long term, but is also mandatory as part of the planning legislation in South Africa [24]. Local knowledge from the community is included in planning processes through community participation programmes. Community input is required for defining local issues and it is widely accepted that community developed solutions are more feasible since they tend to be reasonable, realistic and sustainable [25]. However, these processes are hindered by the fact that communities often do not have access to relevant and up-to-date information, especially spatial information [26]. Even where information is available, it needs to be presented in a language and format that is easily understandable by community members.

Policy makers at local level could use spatial data to assess and develop community facilities such as public transport, health care, schools, and extension of services. The spatial data would in this context be used in an analytical fashion such as "How far has the average person to walk to reach a water tap?" or "How many community members have access to water born sanitation?"

Research on the role of participatory GIS within the services and planning context are available in abundance (see for instance King [27]). Scotch and Parmanto [28] discuss the development of a system designed to monitor and assess community health. It is designed to use information from a variety of sources, and analysis is done through a simple interface. It is user friendly and thus accessible to both health analysts and the non-expert. Elwood and Leitner [29] describe a project in which a variety of local organisations are given access to GIS in order to facilitate community planning. Further examples include the use of GIS to promote health in various forms [30]. Theseira [31], in a study concerned with sharing health related data over the internet, identifies the agencies involved. They include government departments at a local and national level, public health agencies, GIS organisations and the university that initiated the project. Unfortunately, no formal assessment of the usefulness or usability of the system is available.

Many of the abovementioned initiatives show great potential for the management of health. However, all lack adequate documentation of the manner in which they were implemented, and whether this was successful or not, especially in terms of empowerment [32]. This hampers the development and re-use of previous research and implementation experiences.

### Why a web based GIS?

Leitner et al. [33] discuss six models for making GIS available to communities:

• A community based (in house) GIS is an independent node located within a community organisation, usually at its office.

• University-community partnerships. Universities are attempting to assist communities with their spatial information and mapping needs.

• Publicly available GIS facilities in universities and public libraries.

• "Map Rooms" are used by government institutions to provide citizens with maps

• Internet map servers make spatial data available to communities over the internet

• Neighbourhood GIS centres develop when neighbourhoods pool their expertise and resources to provide a central facility that all affiliated community organisations can use.

An internet map server is seen as the most egalitarian method for distributing spatial information [33]. The system being discussed was developed for communities which generally rely on very basic computer systems, such as those provided by a library or internet cafe. Therefore, it was decided to develop a user friendly internet GIS client in combination with an internet GIS server. Web browsers are ubiquitous, which makes it a user-friendly user interface. Furthermore, such a system can be very flexible. Users can either access the system through the internet, through a network or locally. The system could also be distributed on a CD for situations where network connectivity is not available.

### Health related Internet GIS applications

Numerous health-related internet GIS applications are described in available literature. However, most of the systems described operate in a less resource constrained environment than the one described in this paper. Kamadjeu et al. [34] describe how to use Scalable Vector Graphics (SVG) to publish health related maps and graphs on the internet. Blanton et al. [35] have built a spatial database and internet mapping application for real-time rabies surveillance in the United States.

Maclachlan et al. [36] document the development of an internet GIS for the investigation of relationships between health, air quality and socio-economic factors in Canada. A focus group of health professionals was used to test the system and obtain feedback. Results show that the majority of target users can have their needs met on a limited budget.

Goa et al. [37] describe a loosely coupled, interoperable service oriented architecture for online mapping of spatial-temporal disease information based on Open Geospatial Consortium standards. According to the authors, such an infrastructure enhances efficiency and effectiveness of public health monitoring.

### Usability evaluations of internet GIS applications

Research into the usability of internet GIS applications is part of geo-visualisation research. Geo-visualisation can be defined as a field on the use of visual geospatial displays to explore data and through that exploration to answer questions, generate a hypothesis, develop problem solutions, and construct knowledge [38]. Geo-visualisation has attracted a lot of attention from researchers. See for instance Bloemmen et al. [39] for an overview. Slocum et al. [40] argue that visualisations of spatial information should be tested using usability engineering principles. However, the overall usability of maps and map applications has not been fully investigated, or at least not reported in academic research papers [41]

Nivala [41] presents a usability evaluation study of different web mapping sites (Google Maps and its competitors) to find out usability problems with current web maps. Kamarkova et al. [42] describe a method to evaluate the usability of internet GIS applications and apply this method in a case-study. Schimiguel et al. [43] have investigated accessibility of internet GIS applications.

### Open source software

Software is considered open source once it complies with the following characteristics [44]:

• The source code of the product must be made available

• The license allows unlimited redistribution of the product

• The license permits the creation of license free derived works

• The license does not limit how, where or by whom the product can be used

However, the license under which the product has been released is not the most important feature of open source software. To ensure maintenance, support and ongoing development (and through this the success of the project), a community of developers and users working towards the final product has to exist.

The application of open source GIS software provides a number of advantages, especially within a South African context. Fleming [45] notes a number of these:

• Open source GIS software can read and write all GIS data formats

• Open source GIS software will run on multiple platforms

• No license fees are due for open source software

• Freedom to modify the code. This entails vendor independence.

• Money previously spent on license fees outside South Africa is used to develop local skills

Krishnamurthy [46] discusses the advantages of open source software in general. The advantages not mentioned above include:

• Open source software benefits from a large developer and tester base

• Open source software is reliable through peer review

• Flexibility of use

• Support is provided by the community

However, there are some disadvantages associated with using open source software [46].

• There is no formal user support

• A lot of open source projects suffer from version proliferation

• Open source software generally appeals to high end users only

### Open source internet GIS software

Anderson and Moreno-Sanchez [47] find that using open source software has a number of advantages for organisations with scarce resources: no software costs, software tools are easily learned by personnel with general IT background, small software footprints, no need to commit to proprietary software, freedom to extend the software with functionality not present in commercial software and compatibility with existing IT infrastructure. Moreno-Sanchez et al. [48,49] further describe how open source software and open specifications can be used to develop a health related cross-border web-based multimedia spatial information system. The application of open source software and open standards settled issues with regard to differences in health IT infrastructures on both sides of the border.

Holmes [50]describes a spatial data infrastructure (SDI) for Zambia based on open source software. In this paper, the following advantages of using open source software in developing countries are mentioned: open source software can be localised and customised to local languages and cultures, it decreases dependence on first world countries and facilitates a better growing and sustaining of local software companies.

Caldeweyher et al. [51] describe the OpenCIS project. The aim of the OpenCIS project is to provide communities with GIS functionality. This project shows GIS are in demand at community level. OpenCIS is based on UMN MapServer. Kamel Boulos and Honda [52] provide instructions on how to publish health maps on the internet using UMN MapServer and DMSolutions MapLab. The authors state that open source web GIS systems have reached a state of maturity, sophistication, robustness and stability and usability and user friendliness rivalling that of commercial, proprietary GIS and web GIS server products.

### The research

As part of the open source GIS research in the Department of Civil Engineering at the University of Cape Town, the research team investigated whether it is possible to use GIS applications for HIV/AIDS planning and management at local level.

The research question was approached in three ways:

• A prototype system was developed to test the technical feasibility of developing an affordable GIS for local-level service delivery planning.

• Usability considerations were investigated by conducting usability testing on the prototype system.

• Organisational fit and stakeholder perceptions of the system were investigated using semi-structured interviews.

## Methods

Given the newness of the research area and of the technology being investigated, it was considered appropriate to conduct an exploratory case study [53], undertaking hypothesis generation (concerning the feasibility of community-level spatial data access through a web-based open-source GIS) rather than hypothesis testing. The collection of "rich" data from multiple perspectives is prioritised over rigorous system testing. From this perspective, the prototype system is as a tool that promotes discussion and facilitates exploration rather than an artefact in itself.

### Technical feasibility

The technical feasibility of developing the system proposed using open source software was investigated by developing a prototype. The functionality of the prototype is described below. The prototype was subjected to a performance test and was also used to conduct usability tests.

### Functionality of the prototype

The prototype system can be used for the following purposes:

• To access spatial information from a central database by local communities

• To upload spatial information collected by communities

• To access spatial information collected by communities from other government levels

### Local community access to spatial information

The GIS prototype uses choropleth maps to present indicators of access to basic facilities in local communities. These data are also presented in a table.

### Collection of spatial information by communities

The GIS can be used by communities to collect their own data. Through a user friendly interface, end-users can enter attribute data for selected areas. These data are then stored in the central database. This allows the users to analyze their situation, develop a clearer understanding of local needs and gain bargaining power in budget discussions through the display functionality of a GIS. However, one of the key aspects that will require attention in future research is the factor of maintaining data at community level.

### Presentation of spatial information collected by communities to other government levels

Other government levels can gain access to the data entered by end-users at local community level. This can help to verify data at community level and helps to fill data gaps. Communities often have a different experience of the problems they are facing and collecting information that is relevant to the community shows higher decision making levels the priorities of a community.

### Performance measurements

To be able to determine if the system would be able to cope with a reasonable number of users, the system was subjected to an automated load test. During this load test, an increasing number of requests were produced to simulate an increasing number of users. This allowed conclusions to be drawn with regard to the performance of the system. The average response time of the system can be calculated to measure performance.

The performance of the system was assessed using the Apache JMeter software. JMeter is a desktop application which can be used to load test functional behaviour and measure performance of amongst others web applications (both static and dynamic) and databases [54]. The specifications of the hardware used during the performance tests can be found in Table 1. Both the application server and the database were running on the same PC, eliminating network latency effects. Characteristics of the spatial data used during the performance tests can be found in Table 2.

**Table 1:** Specifications of PC used during performance tests

Operating system	Windows XP Professional
CPU	Intel Pentium 4 2.8 GHz

RAM	512 MB

**Table 2:** Data used during performance tests

	Number of features
Provinces	9

Municipalities	30

Sub-councils	28

Sub-places	796

### Usability testing

A usability evaluation was used to investigate the second research question. A usability evaluation involves testing the ability of a system to allow users to complete their tasks effectively, efficiently and enjoyably [55].

The usability evaluation consisted of the following parts:

• Two tasks the users had to perform. The tasks were consistent with the typical use of the prototype. Task one and task two involved identifying an area at the lowest available scale and changing the attribute data of that area. The only difference between these two tasks was the area the participants had to identify. Areas were assigned randomly to participants. The task analysis allows for the identification of problems users may have when using the system. The time the participants needed for each task was measured using a stop watch. There were never more than two people completing the usability tests at once, allowing the facilitator to record accurate times

• Directly after the usability tasks were completed, the participants completed a System Usability Scale (SUS) test in which the participants rate the system to measure the overall usefulness, ease of use and appropriateness of the system. The SUS test is designed to gain a universal and subjective assessment of a system's usability [56].

The results from such tests are generally used to provide comparative values indicating the progress of the system meeting its users needs, and are used to monitor such progress during the development cycle of a system. Formal statistical analysis is therefore not applicable to the results gained from the usability tests, and were not used in the analysis of the results.

### Participants

A convenience sample of users used for tests at the University of Cape Town (UCT). In order to gain a more complete picture of the usability of the prototype, usability testing was also carried out with potential users of the system in a case study of a South African municipality.

The municipality chosen for this case-study was the Overstrand municipality which is located in the south eastern extreme of the Western Cape province of South Africa. The Overstrand municipality has a population of about 80 000 people, but this is growing rapidly, with a population growth rate of 50% from 1996 to 2001, which is expected to continue in the future.

The following potential user groups participated in the testing of the prototype:

• Municipal officials including those who deal with planning of services and service provision, those who deal with community involvement in decision making and those responsible for the ward committees.

• The clinics, represented by clinic personnel, those responsible for the management of clinics and sisters at the clinics

• Community leaders, represented by community organisation members and members of ward councils within the study area

The municipal structure of the study area is representative of other small or medium sized municipalities. The participants were selected based on being representative of those who would use a system as the one described in such a municipality. Together, these groups represent all users working with spatial information in the study area.

All of the local municipalities within the Overstrand District were contacted telephonically (additionally by letter or email if requested), and meetings were arranged with area managers. Only one of the local municipalities within the Overstrand District refused to be involved in the testing. Area managers participated from all the municipalities, community officers and information managers also participated from the district municipality level. The sample of municipal officials represents the potential functional users and managers of the system described. A list of all the clinics in the district was obtained from the municipality, and after obtaining permission from the district clinic, each local clinic was contacted and a staff member was asked to be available for testing at a predetermined time. The majority of the clinics in the area responded positively and participated in the study. Due to the large number of community organisations, not all were contacted, and only those that were thought to have interest in the system and testing were contacted, few of which chose to participate in the study.

The user profile of the participants is summarized in Table 3. Participants received no training prior to the tests. They were introduced to the system, and given an introduction to the purpose and goals of it.

**Table 3:** Summary of user profile of participants in usability analysis

	**Count**	**Percentage**
Sample size	30	100

**User group**		

Clinics	7	23,33

Community	4	13,33

Convenience	10	33,33

Municipality	9	30

**Level of computer use**		

Coding	4	13,33

Internet	17	56,67

Word-processing	9	30

**Education level**		

High school	5	16,67

Tertiary	25	83,33

**Gender**		

Female	18	60

Male	12	40

**Age**		

20–29	10	33,33

30–39	6	20

40–49	10	33,33

50–59	4	13,33

### Semi-structured interviews

To gain more insight into the appropriateness of the system in terms of existing structures (the third research question) interviews with potential users in the South African municipality described above were conducted. An semi-structured interview is an interview without a predefined format. The interviewer however did prepare some key questions. It is a widely used method of finding out what users want [57]. The municipal officials, clinic personnel and community representatives who participated in the usability test, participated in the interviews as well.

## Results and discussion

### Technical feasibility

The design and implementation of the prototype show that it is feasible to develop a system such as the one described in this paper using open source GIS software. This result is consistent with findings of other authors [47-51,58].

#### Components of the prototype

**Figure 1:**
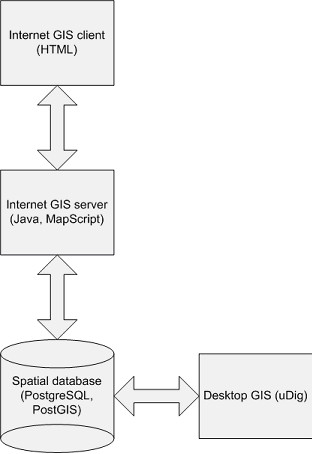
**Components of the GIS prototype**. The prototype system was developed as a series of components. End-users access the system by logging in to the internet GIS client. The internet GIS client connects to the internet GIS server. The internet GIS server contains all of the application logic. It was developed in Java. MapScript, the scripting language for MapServer, was used to implement the GIS functionality. The spatial data are stored in a PostGIS spatial database. PostGIS is the spatial extension to the PostgreSQL relational database management system. Administrators use a desktop GIS application (for instance uDig) to manage the spatial database.

**Figure 2:**
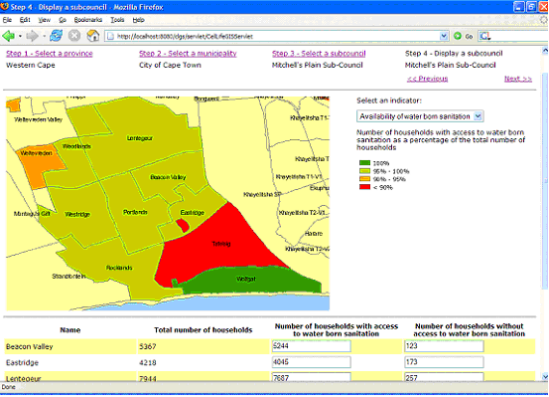
**Screenshot of the internet GIS client**. End-users access the system by logging in to the internet GIS client. The client runs inside a web browser, as illustrated, and requires no additional software or plug-ins.

The prototype system was developed as a series of components (see Figure 1). End-users access the system by logging in to the internet GIS client (see Figure 2 for a screenshot). In order to facilitate an easy installation and to minimize the footprint on the client computer, it was decided to develop a thin HTML client. This client runs inside a web browser and requires no additional software or plug-ins.

The internet GIS client connects to the internet GIS server. This internet GIS server contains all the application logic. It was developed in Java, a platform independent object oriented programming language. MapScript, the scripting language for MapServer, was used to implement the GIS functionality. MapServer is a development environment for building spatially enabled internet mapping applications [59]. The spatial data are stored in a PostGIS spatial database. PostGIS is the spatial extension to the PostgreSQL relational database management system [60]. This database contains the geographical data that MapServer uses to produce maps. Administrators use a desktop GIS application (for instance uDig [61]) to manage the spatial database.

Both MapServer and PostGIS are well documented and the communities which have developed around these products provide active support. According to Krishnamurty [46], open source software appeals to high end users only. Anderson and Moreno Sanchez [47] however argue that open source GIS software tools are easily learned by personnel with a general IT background and Kamel Boulos and Honda [58] show how health maps can be published on the internet by users with no prior technical experience in web GIS or internet GIS servers. Through this research we have experienced the latter.

Modification of the source code of the software applied proved unnecessary, so no conclusions can be drawn with regard to this subject. And although a lot of different versions of the tools applied are available, version proliferation did not cause problems. Documentation and support of the community was abundant for the versions used.

#### Cost effectiveness

An internet GIS application relieves communities of much of the hardware, software, data, expertise, and maintenance costs associated with other forms of GIS delivery [36,62]. The application of open source software alleviates the organizations involved of the license and maintenance fees required for proprietary GIS software. And since open source GIS software tools are easily learnt by personnel with a general IT background [47], training costs are minimised.

A detailed cost-benefit analysis of the prototype system was beyond the scope of this research. However, it can be seen that the choice of an open source web based system effectively minimises barriers to end-user access, while the benefits of community-based GIS extend beyond efficiency and effectiveness to community empowerment [63].

#### Data

The data presented in the GIS originate from the census conducted by Statistics South Africa in 2001 [64]. Due to confidentiality issues, the data of the 2001 census were aggregated. The lowest level at which the census results were released was the second tier of the spatial hierarchy, the sub-place [65].

Sub-places are aggregated from the first tier, the enumeration area. They are categorized and named according to the name of the suburb, electoral ward, village, farm or informal settlement [64], covering populations of at least 500 people [65]. The sub-places are displayed within the borders of the wards, or sub-councils, as defined by the municipality responsible for the area.

In the prototype, data related to access to piped water, water born sanitation and electricity are available. Other datasets could easily be added. However, using more direct indicators of the health situation of the population (for instance the number of HIV infected people or the number of people having AIDS) was not possible because of confidentiality issues.

#### Choropleth maps

The GIS prototype displays a choropleth map, a map that illustrates the rate of a phenomenon over a certain area. Choropleth mapping is an effective tool for viewing patterns within a data set and are commonly used for visualizing socio-economic patterns, disease and various other human geographic variables. In addition to these characteristics choropleth maps have the added advantage that they are familiar to a wide audience [66], increasing the appeal and usability of the GIS prototype.

Boscoe and Pickle [66] identify a number of desired characteristics of choropleth maps, including a high degree of spatial resolution, allowing for more details and potentially more patterns to be presented on the map. A second desirable feature of choropleth maps in that the population and area sizes are homogenous, so that the number of observations is equally spread over equal areas on the map. Failing this, the areas with the smallest populations, and the smallest areas will show extreme values. In order to reduce this, the areas were identified through the use of wards, which have approximately the same population size.

Unfortunately, integrating all the desirable features of a choropleth map on one map is not a possibility [66], and it is thus one of the aims of the case study to determine which features are priorities to the users of the GIS prototype. The initial aim of the prototype was one of simplicity, and thus a low spatial resolution was selected, as well as areas that would be familiar to the users.

In addition to the choropleth maps, a table containing the attribute data is available to the user through the interface.

#### Performance

**Figure 3:**
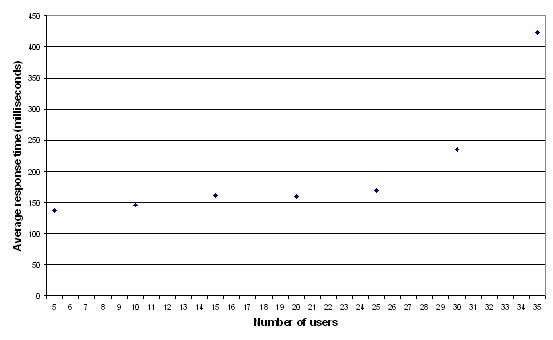
**Average response times application server**. This graph shows the average response time of the application server in milliseconds for a given number of concurrent users. These users have been simulated using Apache JMeter. Between 0 and 25 concurrent users, the average response time hardly increases. With more than 25 users, the average response time starts to increase. This is caused by a queue of requests which increases. This indicates the system is capable of handling 25 concurrent users.

Figure 3 shows the average response times of the application server with an increasing number of concurrent users. It shows that average response times start to rise when about 25 users are concurrently using the system. This is a queue which is developing. It indicates the system on the hardware used during the tests can cope with 25 concurrent users. Since the anticipated number of concurrent users is ten, the performance tests showed that the system will be able to cope with the anticipated number of concurrent users. Ten was chosen as a realistic number of concurrent users, because the access to computer hardware is limited within communities.

#### Discussion of method

Developing a prototype system provides valuable insight into the feasibility of certain solutions. The prototype facilitated other research activities, such as usability analysis. However, developing a prototype can be time consuming. Furthermore, it is often tempting to deploy a prototype in a production environment, but this often not a good idea since different requirements apply for a production system.

Automated load tests using Apache JMeter provide valuable insight in the behaviour of a system under heavy load.

### Usability

The results of the usability analysis are summarized in Table 4. This table shows the average, time taken for the completion of task one and task two, the average difference in time taken for the two tasks and the average SUS scores. Table 4 also shows the ranges for these variables. The information is presented for all participants and broken down according to user group, age, computer literacy, education and gender.

**Table 4:** Summary of results of usability analysis.

	**Average time task one (minutes)**	**Average time task two (minutes)**	**Difference average time task one and two**	**Average SUS**
Sample	3.27 (0.47–8)	0.83 (0.1–2)	2.44	68.50 (35–97.5)

**User group**				

Clinics	3.24 (1.6–5)	1.16 (0.8 – 1.5)	2.09	64.64 (55–72.5)

Community	5.63 (2.5–8)	1.23 (0.9–2)	4.40	46.88 (35–75)

Convenience	1.83 (0.47–3)	0.32 (0.1–1.12)	1.51	78.75 (45–97.5)

Municipality	3.83 (2–6)	0.95 (0.45–1.6)	2.89	69.72 (62.5–85)

**Level of computer use**				

Coding	2.92 (1.16–5)	0.75 (0.1–1)	2.17	66.88 (35–95)

Internet	2.93 (0.47–5)	0.63 (0.15–1.4)	2.30	72.06 (45–97.5)

Word-processing	4.07 (1.6–8)	1.23 (0.45–2)	2.84	62.50 (35–72.5)

**Education level**				

High school	4.90 (2–8)	1.33 (1–2)	3.58	56.50 (35–72.5)

Tertiary	2.94 (0.47–6)	0.73 (0.1–1.6)	2.22	70.90 (35–97.5)

**Gender**				

Female	2.95 (0.47–7)	0.87 (0.1–2)	2.08	66.53 (35–97.5)

Male	3.75 (0.5–8)	0.76 (0.2–1.6)	2.99	71.46 (42.5–92.5)

**Age**				

20–29	2.52 (0.47–8)	0.39 (0.1–1)	2.13	72.75 (42.5–95)

30–39	4.29 (1.6–7)	1.21 (0.25–2)	3.08	67.50 (35–97.5)

40–49	3.51 (1.6–5)	1.02 (0.45–1.5)	2.49	64.50 (35–75)

50–59	3.00 (2–5)	0.88 (0.5–1)	2.13	69.38 (55–85)

The table shows the average time taken to complete task one and task two, the difference in the average time taken to complete task one and two and the average SUS. The range (minimum and maximum) of these indicators is shown between brackets.

All the participants completed the tasks successfully. The results of task one and task two were used to obtain a measure of the learnability of the system. The average time taken to complete task one is 3.27 minutes. The average time taken to complete task two is 0.83 minutes. The difference in time taken between task one and task two is reasonably large, indicating that the system is easy to learn. The average SUS score of 68.5 is consistent with SUS tests performed in contexts that may be related to this research [67,68]. Even in its early stages, the prototype shows great potential.

The results of usability tests conducted among the convenience sample at the University of Cape Town (UCT) shows a high degree of usability for the system, with System Usability Scores (SUS) averaging close to 80 and task times averaging just over two minutes to find the first area, and well under a minute for the second, improving with the time taken for similar tasks and indicating that the system is generally user friendly and easy to learn.

The results of usability tests in the case-study show a general increase in the time taken for each task, as well as more critical comments than the results gained from the convenience sample. However, task times once again decreased as they did with the convenience sample indicating a high degree of learnability. The critical comments were consistent across the sample, and thus have provided a clear direction for further participatory development of the prototype. Differences in time taken to complete the tasks and SUS may be explained by the fact that participants in the convenience sample are on average younger (see Table 5).

**Table 5:** Sample characteristics versus user group

**User group**	**No. participants**	**Average age**	**% males**	**% females**	
Clinics	7	46	0	100	

Community	4	36	50	50	

Convenience	10	27	30	70	

Municipality	9	42	77.78	22.22	

Sample	30	37	40	60	

					

**User group**	**% word processing**	**% internet**	**% coding**	**% high school**	**% tertiary**

Clinics	57.14	42.86	0	28.57	71.43

Community	50	0	50	50	50

Convenience	10	80	10	10	90

Municipality	33.33	55.56	11.11	0	100

Sample	33.33	53.33	13.33	16.67	83.33

Education level does seem to influence the user's performance on the system, as well as the user's perception of the system, with time taken for the task generally longer for the high school graduates than those with a tertiary education, and the SUS scores being lower. This is a result of the general skills gained through education and is an expected outcome.

Previous experience in computer use has a minimal effect on the system's usability, with those experienced in coding and internet use performing better than those with word-processing experience only. It is interesting that those with experience in using the internet slightly out performed those with programming experience. This illustrates that minimal computer skills, not significantly different from the skill set required for general internet use, are required for the use of the GIS prototype.

Age and gender show minimal variations, those at the two extremities of the age groups performed most effectively, illustrating that age is non influential in the usability of the system. The female user group performed generally better than the male counterparts.

#### Discussion of method

Usability testing methods allow for a subjective means of measuring the usability of the system. Such measurements allow for the comparative analysis of various aspects affecting usability. The use of this measurement may also be compared throughout the lifecycle of a system. The measurement is however, limited to being compared within the context of the system and is not comparable across different systems. For a prototype, test results function as a broad-brush indicator of potential usability of a full system, at a conceptual rather than detail level. Usability testing of a prototype may also help to identify consistent problem areas to be addressed in a full system.

It should be recognised that the convenience sample of participants, both in the university student group and in the municipal user group, introduces bias into the sample on the basis of age, education level, motivation (participants who self-select are likely to be supportive of system objectives) and familiarity with computers and internet use. Results from this group would have limited validity in generalisation to all potential users. However, as the purpose of usability testing in this research is not to provide precise measurement (which in any case would be inappropriate for a prototype system), but rather to demonstrate the potential for development of a usable system based on the concept of the prototype, the convenience sample was considered sufficient.

### Semi-structured interviews

The comments made by the participants in the semi-structured interviews are summarized in Table 6. The area managers and ward councillors of the Overstrand municipality, who are responsible for providing an environment of participatory development, do not currently make use of any formal information systems for the transfer of information internally or externally, and only informal means of information transfer are employed. Examples of information sharing and transfer provided include letters or meetings. This has been shown to limit access to information, and the ability to self-govern, especially for marginalized groups.

**Table 6:** Summary of usability testing post test interview results, user group communities/clinics.

**Question**	**User group municipalities**	**User group communities/clinics**
Should GIS be used by communities	• Yes, but concerns over how it may work	• Yes
	• Yes, through NGO's	• Knowledge about local conditions
	• Yes very interested	• They need all the help they can get
	• Yes, in libraries	
	• Yes, real estate	

		

Benefits – municipality	• Budgeting • Information	• Planning • Knowledge of facilities
	• Awareness	• Understanding of services by community
	• May pose problems, but should be beneficial	• Understanding of local conditions
	• Less phone calls	• Identification of problems
	• Relief on municipal staff	• Home based carer management
	• Updated data	
	• Communication with municipalities	
	• Informed decisions	
	• Co-operation	
	• Local knowledge	
	• Self governance	

		

Benefits – community	• Information availability	• Better living conditions
	• Transparency of government information	• Service improvements
	• improved contribution to planning and development Understanding of planning and priorities	• Insight
	• Better informed	• Engage with municipalities and challenge their decisions
	• Information accessibility	• Upliftment
	• Knowledge	• Education
	• Ability to articulate needs	
	• Preparation for community meetings	

		

Hinder	• Skills	• Computer literacy
	• Political parties	• No access to internet
	• Resources	• Knowledge
	• Cost	• Understanding
	• Facilities	• Misuse
	• Access to resources	• Skills
	• Resources	• Resources
	• Distribution	• Map reading
	• Accessibility	• Computer literacy
	• Management	

		

General impression	• Positive, but with reservations	• Good, happy to be involved in the development
	• Positive, but will need facilitation	• Promising
	• Could be of benefit	• Good, Creating awareness and sharing knowledge
	• Positive, there is a need for mechanisms such as this	• Good, but needs to be simplified
	• Positive, understanding and awareness	• Positive
	• Generally positive	

Repetitive answers have been omitted

The respondents do however see the potential for the use of GIS as a communication tool within their constituency and are generally positive about the use of GIS for these purposes. They do note hindrances to the implementation of a system and the sharing of information through a system such as the one proposed.

The issues noted may be separated into three groups: skills of the users, resource limitations and control issues. Skills shortages were noted in terms of computer literacy, map reading ability, and general understanding (conceptual understanding of the system). These hindrances are addressed through the system's relative simplicity and learnability. Resource limitations are a common problem across all municipalities. What many of the participants did not realise is that the infrastructure required for the system was in place already in most cases (computers and phone lines), and no substantial investment in this would be required. The control issues relate to management of the system, potential political interference, misuse of the information and exclusion of certain groups. These are all realities, and perhaps where the most work will be required in the implementation phase of the system. Structures will have to be implemented alongside the system to ensure data integrity, protection from misuse or interference, and ensuring fair access to the information produced by the system.

The issues above are counteracted by many positive outcomes that can be provided to the community and the municipality. These include updated information, local knowledge, more informed communities and decision making, awareness and transparency of the local government. Furthermore the municipality is in the process of implementing commercial software for internal use, thus providing a base set of data from which the prototype could gain. The prototype could also play an important role in terms of serving the satellite municipal offices who are not involved in the implementation or use of the commercial software.

#### Discussion of method

The use of semi-structured interviews allows for three advantages. Firstly using this method allows for the gaining of buy-in of gatekeepers and managers. This is critical in the implementation, or planning of implementation of any system within any organisation. Secondly, it allows for the determination of the higher structures that may determine or limit the implementation of the system. Finally, and most critically the process allows for the gaining of information regarding existing information systems and communication structures.

The lack of structure in the interviews can allow for the subject to be manipulated by the interviewee, and thus it is critical for the interviewer to maintain focus on the subject of the interview and ensure that the required information is gained through the discussion. In this research, focus was maintained by arranging interviews around a system demonstration, and by involving interview subjects in usability testing.

## Conclusion

The aim of this research was to investigate the possibilities of using GIS in a local under-resourced context to establish if communities can be empowered to collect and analyse their own data sets. This study was the first step to assess the technical and usability aspects of such a system.

The research conducted confirms that it is possible to use open source GIS software to develop a user-friendly GIS application, containing functionality to provide local communities with spatial information. Performance is sufficient and the choice of a web-based open source system negates the need for specialised end-user software. This promotes equitable access in accordance with government policy.

The results of the usability tests show a high degree of learnability and an average to good usability for the system. The critical comments provide a good point to work from in terms of further development of the prototype.

The semi-structured interviews conducted within the frame of the case-study show spatial information is in demand at local community level. But the case-study also shows there are some serious information management and organisational issues which need to be solved before an implementation of the system described in this article will be successful.

## Competing interests

The authors declare that they have no competing interests.

## Authors' contributions

BV conceived of the study, participated in its design and coordination, developed the prototype and helped to draft the manuscript. AR conducted the case study and usability tests and helped to draft the manuscript. ML provided the background information and helped to draft the manuscript. UR conceived of the study, provided the funding and participated in its design and coordination and helped to draft the manuscript. All authors read and approved the final manuscript.
